# No effect of ablation of surfactant protein-D on acute cerebral infarction in mice

**DOI:** 10.1186/1742-2094-11-123

**Published:** 2014-07-19

**Authors:** Kate L Lambertsen, Kamilla Østergaard, Bettina H Clausen, Søren Hansen, Jan Stenvang, Stine B Thorsen, Michael Meldgaard, Bjarne W Kristensen, Pernille B Hansen, Grith L Sorensen, Bente Finsen

**Affiliations:** 1 Department of Neurobiology Research, Institute of Molecular Medicine, University of Southern Denmark, JB Winsloewsvej 25, 2, DK-5000 Odense C, Denmark; 2 Department of Cardiovascular and Renal Research, University of Southern Denmark, DK-5000 Odense, Denmark; 3 Department of Cancer and Inflammation Research, Institute of Molecular Medicine, University of Southern Denmark, DK-5000 Odense, Denmark; 4 Department of Neurology, Odense University Hospital, DK-5000 Odense, Denmark; 5 Department of Pathology, Odense University Hospital, DK-5000 Odense, Denmark; 6 Institute of Veterinary Disease Biology, Faculty of Health and Medical Sciences, University of Copenhagen, DK-2220 Copenhagen, Denmark

**Keywords:** Stroke, Middle cerebral artery occlusion, Collectins, Tumor necrosis factor, Microglia, Macrophages, Phagocytosis, Plasticity

## Abstract

**Background:**

Crosstalk between the immune system in the brain and the periphery may contribute to the long-term outcome both in experimental and clinical stroke. Although, the immune defense collectin surfactant protein-D (SP-D) is best known for its role in pulmonary innate immunity, SP-D is also known to be involved in extrapulmonary modulation of inflammation in mice. We investigated whether SP-D affected cerebral ischemic infarction and ischemia-induced inflammatory responses in mice.

**Methods:**

The effect of SP-D was studied by comparing the size of ischemic infarction and the inflammatory and astroglial responses in SP-D knock out (KO) and wild type (WT) mice subjected to permanent middle cerebral artery occlusion. SP-D mRNA production was assessed in isolated cerebral arteries and in the whole brain by PCR, and SP-D protein in normal appearing and ischemic human brain by immunohistochemistry. Changes in plasma SP-D and TNF were assessed by ELISA and proximity ligation assay, respectively.

**Results:**

Infarct volumetric analysis showed that ablation of SP-D had no effect on ischemic infarction one and five days after induction of ischemia. Further, ablation of SP-D had no effect on the ischemia-induced increase in TNF mRNA production one day after induction of ischemia; however the TNF response to the ischemic insult was affected at five days. SP-D mRNA was not detected in parenchymal brain cells in either naïve mice or in mice subjected to focal cerebral ischemia. However, SP-D mRNA was detected in middle cerebral artery cells in WT mice and SP-D protein in vascular cells both in normal appearing and ischemic human brain tissue. Measurements of the levels of SP-D and TNF in plasma in mice suggested that levels were unaffected by the ischemic insult. Microglial-leukocyte and astroglial responses were comparable in SP-D KO and WT mice.

**Conclusions:**

SP-D synthesis in middle cerebral artery cells is consistent with SP-D conceivably leaking into the infarcted area and affecting local cytokine production. However, there was no SP-D synthesis in parenchymal brain cells and ablation of SP-D had no effect on ischemic cerebral infarction.

## Introduction

Surfactant protein-D (SP-D) is a C-type collectin mediating various effector mechanisms, such as opsonization of pathogenic microorganisms and cellular debris, chemotaxis of phagocytes [1], activation of phagocytosis [2,3] and modulation of toll-like receptor (TLR) activity [4,5]. SP-D knock out (KO) mice display increased lung inflammation, including increased lung levels of pro-inflammatory cytokines, metalloproteinases and oxidant species [3,6,7]. Besides important roles in pulmonary innate immunity, SP-D is also involved in systemic lipid homeostasis [8,9]. The major site of SP-D synthesis is the pulmonary epithelium and other epithelia [10], however, SP-D synthesis also takes place in vascular smooth muscle [11] and vascular endothelial cells [9,12]. SP-D has been demonstrated to affect vascular pathology and to be pro-atherogenic in mice [9].

Studies of experimental and clinical stroke indicate that the complement system contributes to ischemic injury [13-15]. The collectin mannose-binding lectin (MBL) has been shown to play a central pathogenic role in ischemic injury through deposition and complement activation on ischemic endothelium [16-18]. SP-D does not activate complement, yet, serum SP-D levels were recently shown to correlate to dementia and augmented mortality [19], raising the possibility that SP-D may affect neurodegenerative and inflammatory processes in the brain. Suggested cellular receptors for SP-D include TLR2 and TLR4 [4], which are upregulated after induction of focal cerebral ischemia [20] and the myeloid inhibitory immunoreceptor Src homology 2 domain-containing protein tyrosine phosphatase substrate-1 (SHPS-1/SIRPα) [21,22]. SHPS-1/SIRPα is expressed by neurons and phagocytic cells and is recognized to be involved in neuroprotection in experimental stroke in mice [23]. Plasma proteins leak into the ischemic brain both after reperfusion-ischemia, and permanent focal cerebral ischemia, where leakage takes place through collaterals in the peri-infarct. We therefore hypothesized that SP-D might leak into the ischemic area either from the circulation or from the deranged endothelium and reduce infarction after experimental stroke in mice.

## Materials and methods

### Animals

For the infarct volumetric and glial/leukocyte analyses we used young adult (six to nine weeks), male SP-D KO mice [24], crossed > ten times onto the C57BL/6 N background [25]. Mice were kept as a colony at the Biomedical Laboratory, Institute of Molecular Medicine, University of Southern Denmark. The mice have previously been shown to be deficient in SP-D mRNA and protein expression [24]. Male, age-matched, wild type (WT) C57BL/6 N mice were purchased from Charles River (Wilmington, MA, USA). Other analyses were performed on six- to nine-week old male C57BL/6 J mice or NMRI mice, purchased from Taconic (Ry, Denmark). The NMRI mice (non-inbred) were included for comparison with C57BL/6 mice for vasculature analysis. Mice were housed under diurnal lighting conditions with free access to food and water. The experimental procedures were approved by the Danish Animal Ethical Committee (J number 2005/561-1068 and 2011/561-1950).

### Focal cerebral ischemia

Focal cerebral ischemia was induced by permanent occlusion of the distal part of the left middle cerebral artery (pMCAO) as previously described [26]. SP-D KO and WT mice were randomized to three experimental groups: one group of unmanipulated control mice and two groups of mice that were subjected to pMCAO and allowed either one day or five days post-surgical survival. For analysis of plasma TNF, C57BL/6 mice were allowed 30 minutes, and 1, 2, 4, 6, 12 and 24 hours survival after pMCAO. Furthermore, C57BL/6 mice with five days survival were included for immunohistochemical detection of SP-D.

Mice were anaesthetized by subcutaneous (sc) injection of a mixture of Hypnorm (fentanyl citrate 0.315 mg/ml and fluanisone 10 mg/ml, Jansen-Cilag A/S, Birkerød, Denmark), Stesolid (5 mg/ml diazepamum, Dumex) and distilled water (1:1:2) (0.21 ml/10 g body weight). For post-surgery analgesia, mice were injected with Temgesic (0.001 mg/20 g buprenorphinum sc, Schering-Plough A/S, Ballerup, Denmark) three times with an 8-hour interval starting immediately after surgery. Mice were allowed to recover from surgery in a recovery room at 28°C. Mice allowed five days survival were returned to the conventional animal facility after 24 hours.

### Mouse tissue processing

#### Fresh frozen brain tissue

Mice were killed by cervical dislocation. Upon removal, brains were frozen in CO_2_-snow and cut into 6 series of 30-μm-thick sections. Every 12th section was placed in an RNase free Eppendorf tube for RNA-extraction, reverse transcription and qPCR analysis of whole brain. Tissue was stored in sealed boxes at −80°C until further processing. Lung and spleen tissue, intended for standard curves in qPCR analyses, was obtained from a subset of mice.

#### Paraformaldehyde fixed brains

Mice to be used for immunohistochemistry for SP-D were overdosed with Pentobarbital (Den kgl. Veterinær- og Landbohøjskoles Apotek, Copenhagen, Denmark) and perfused through the left ventricle with 5 ml chilled 0.15 M Sorensen’s phosphate buffer (So-PB, pH 7.4) followed by 20 ml chilled 4% paraformaldehyde (PFA) in 0.15 M So-PB, pH 7.4 [27]. Brains and lung tissue were post-fixed in 4% PFA for 1 hour, followed by 1% PFA overnight, and rinsing in PBS before embedding in paraffin. Paraffin sections were prepared as 10-μm-thick sections mounted on Super Frost slides (Hounisen, Skanderborg, Denmark).

#### Preparation of plasma for TNF analysis

At the time of decapitation, blood was collected into EDTA coated Eppendorf tubes. Samples were spun twice at 3,000 × g for 10 minutes and supernatants collected and stored at −80°C, until further processing.

#### Preparation of plasma for SP-D analysis

Blood was sampled by eye vein puncture and collected into EDTA coated Eppendorf tubes for SP-D plasma analysis. Blood was obtained from naïve C57BL/6 mice and from pMCAO mice immediately before surgery or decapitation.

### Infarct volumetric analysis

One series of brain sections from mice that had survived one or five days was stained with toluidine blue and the direct infarct volume was estimated using the Cavalieri principle for volume estimation [26,28]. The volume of the infarct (V_total_) was calculated using:

$$V_{\mathrm{total}}=\sum P\;\cdot \;t\;\cdot \;a_{\mathrm{po}\mathrm{int}}$$

where ∑P is the total number of points hitting the infarct, t is the mean distance between sections, and a_point_ represents the area per point [28]. The point-counting was performed using the Computer Assisted Stereological Test (CAST) GRID microscope-system (Olympus Denmark A/S, Ballerup, Denmark). In addition, the volume of the contralateral cortex, the non-ischemic ipsilateral cortex, and the volume of the infarct spanning from 1,080 μm anterior to 1,080 μm posterior of the anterior commissure was compared in SP-D KO and WT mice at day 1 after pMCAO using a method of indirect infarct volume estimation [29,30].

### Immunohistochemistry for murine CD11b, CD45 and Gr1

#### Antibodies

The primary antibodies used for detection of CD11b immunopositive (CD11b^+^) microglia/macrophages, CD45^+^ microglia/leukocytes, and Gr1^+^ polymorphonuclear leukocytes (PMNs) and macrophages, consisted of monoclonal rat-anti-CD11b (clone 5C6, MCA711), rat-anti-CD45 (clone IBL-3/16, MCA1388), and rat-anti-Gr1 (clone RB6-8C5, MCA2387GA), respectively, which were obtained from Serotec, Kidlington, UK. Rat IgG1 or IgG2_b_ (Nordic Biosite, Sweden) were used as isotype controls. Control stained sections where the primary antibody was replaced with either buffer or isotype control were devoid of specific staining.

#### Staining procedure

##### CD11b staining

The fresh frozen sections were fixed 2 minutes in 4% formalin, followed by 2 minutes in 50% acetone, 2 minutes in 100% acetone, and 2 minutes in 50% acetone [27]. Following fixation and air drying, sections were rinsed in Tris-buffered saline (TBS) + 1% Triton, pH 7.4, for 30 minutes and treated with 10% fetal calf serum (FCS) in TBS for 30 minutes at room temperature (RT). Next, sections were incubated overnight at 4°C with primary rat-anti-CD11b diluted in TBS and 10% FCS (1:600). The next day, sections were rinsed 3 × 15 minutes in TBS + 1% Triton at RT and incubated for 1 hour at RT with the secondary biotinylated goat anti-rat IgG (Vector BA1400) diluted 1:200 in TBS and 10% FCS, followed by rinsing 3 × 15 minutes in TBS + 1% Triton, and incubation for 1 hour at RT with streptavidin-horse radish peroxidase (HRP) (GE Healthcare, Chalfont St Giles, UK) diluted 1:200 in TBS and 10% FCS. Sections were thereafter rinsed 3 × 15 minutes in TBS and developed in 0.05% diaminobenzidine (DAB) and 0.033% H_2_O_2_ in TBS at RT. Finally, sections were coverslipped with Depex.

##### CD45 staining

The fresh frozen sections incubated with the rat-anti-CD45 IgG were processed as described above. After incubation with rat-anti-CD45 (1:50), the sections were rinsed 3 × 15 minutes in TBS, incubated with alkaline phosphatase conjugated anti-rat IgG (Sigma A8438) diluted 1:50, rinsed 3 × 15 minutes in TBS, followed by a 15 minute rinse in 10 mM Tris-HCl buffer (pH 9.5), and development in a Tris-HCl-MgCl_2_ buffer (pH 9.5) containing nitro-blue tetrazolium chloride and 5-bromo-4-chloro-3-indolyl phosphate yielding a bluish-black reaction product [27]. Sections were finally rinsed in TBS followed by H_2_O and coverslipped with Aquatex (Merck Millipore, Darmstadt, Germany).

##### Gr1 staining

The staining for Gr1 (1:200) was performed on sections from PFA-fixed brains. The procedure was as described for CD11b using DAB for the development. To allow distinction of nuclear characteristics, sections were counterstained with toluidine blue prior to dehydration and coverslipping with Depex.

### Immunofluorescence staining for glial fibrillary acidic protein

Fresh frozen sections intended for fluorescence staining for astroglial glial fibrillary acidic protein (GFAP) were fixed in 4% PFA overnight at 4°C, air dried and processed as described in Clausen *et al*. [31] using a rabbit anti-cow GFAP antibody (DakoCytomation A/S, Glostrup, Denmark) and a Alexa Fluor® 488-conjugated goat anti-rabbit antibody (Invitrogen, Life Technologies Europe BV, Nærum, Denmark). Sections incubated with buffer or rabbit serum (DakoCytomation) were devoid of signal.

### Microdissection of cerebral arteries in mouse

The deeply anesthetized mice were perfused with ice cold isotonic sodium chloride, and the brain was dissected and placed in ice cold physiological salt solution (in mmol/L: NaCl 115, NaHCO_3_ 25, MgSO_4_ 1.2, K_2_HPO_4_ 2.5, CaCl_2_ 1.3, glucose 5.5, and HEPES 10). Posterior communicating arteries and middle cerebral arteries were isolated under a stereomicroscope and frozen in liquid nitrogen along with the aorta. RNA was extracted as described below.

### Quantitative real-time PCR on cerebral arteries and mouse brain

Mouse arteries from C57Bl/6 (inbred) and NMRI (non-inbred) mice were crushed in a mortar and RNA was isolated using TRIzol reagent from Invitrogen (Carlsbad, CA, USA) and reverse transcribed using Superscript and oligo (dT). RNA extraction and cDNA synthesis of brain tissue were performed as previously described [32]. Real-time detection of SP-D in brain homogenates and isolated mouse arteries was performed on an iCycler using SYBR Green as the fluorescent reporter molecule as previously described [33] and the following primer sets:

1) sense: AAGGGTGATCCAGGTTTGCCA and antisense: GAGGTCCACTTAGTCCACGTTCT (exon 2-3-4),

2) sense: AACGTGGACTAAGTGGACCTCC and antisense: AGCACCTACTTCTCCTTTGGGC (exon 3-4-5),

3) sense: GGAGAAGTAGGTGCTCCTGGC and antisense: GCATTCCCTGGGGCTCCTTG (exon 5),

4) sense: CCTGGAGACAGAGGAATCAAAGGT and antisense: CAGGGAACAATGCAGCTTTCTGA.

Real-time detection of CD11b, TNF, hypoxanthine phosphoribosyl-transferase 1 (HPRT1) and glyceraldehyde 3-phosphate dehydrogenase (GAPDH) was based on fluorescent detection generated by TaqMan probes and primer sets using the following primers and probes:

CD11b (sense: CGGAAAGTAGTGAGAGAACTGTTC, antisense: CTTATAATCCAAGGGATCACCGAATTT and probe: TCTGTGATGACAACTAGGATCTTCGCAGCA), and probe: TGCACTACAGGCTCCGAGATGAACAACAA),

TNF (sense: TGGCCTCCCTCTCATCAGTTC, antisense: CCACTTGGTGGTTTGCTACGA and probe: TGGCCCAGACCCTCACACTCAGATCATC),

HPRT1 (sense: GTTAAGCAGTACAGCCCCAAAATG, antisense: AAATCCAACAAAGTCTGGCCTGTA and probe: AGCTTGCTGGTGAAAAGGACCTCTCGAAGT),

GAPDH (sense: TCAAGCTCATTTCCTGGTATGACA, antisense: CTTACTCCTTGGAGGCCATGTAG and probe: TCCACCACCCTGTTGCTGTAGCCG) as previously described [30,32,34].

Standard curves were generated from a five-fold diluted series of cDNA derived from either spleen (CD11b, TNF, HPRT1, GAPDH) or lung tissue (SP-D). Data were normalized to the reference genes HPRT1 or GAPDH. All values were calibrated to a pool of cDNA from naïve C57BL/6 N mice. Less than two-fold increases in transcript levels were not subjected to statistical analysis [32]. SP-D data are presented as 2dd-Ct values.

### Immunohistochemistry for SP-D in mouse brain

In addition to purchasing a commercially available rabbit anti-mouse SP-D (AB3434, lot number: NG1734281, Merck Millipore) antibody, we tested a panel of 20 monoclonal antibodies made in-house by immunizing SP-D KO mice with recombinant SP-D for their specificity towards murine SP-D. Antibodies were tested on sections from paraffin embedded and frozen tissue samples using different fixation protocols. None of the above antibodies yielded specific-like staining

### ELISA for SP-D in plasma

Mouse SP-D ELISA was carried out as described previously [35]. The validated and calibrated in-house ELISA takes advantage of two monoclonal antibodies recognizing different epitopes on mouse SP-D. The detector antibody is biotinylated and detected with streptavidin-HRP. Readouts were performed with o-phenylenediamine dihydrochloride (OPD) as substrate and measured at 492 nm with subtraction of 600-nm background-signal. Samples of plasma were diluted 1:5 and measured in duplicates. The minimal detection limit of the ELISA was 0.9 ng/ml, which after dilution of plasma samples resulted in a detection limit of 5 ng/ml. Sample concentrations below detection limit are referred to as < 5 ng/ml.

### Proximity ligation assay for TNF in plasma

Proximity probes were prepared using the Proseek probemaker kit (Olink Bioscience, Uppsala, Sweden) according to the manufacturer’s instruction and all the mentioned reagents were contained within the kit. In brief, 1 μL of conjugation buffer was mixed with 10 μL of affinity-purified polyclonal TNF antibody (1 mg/mL) (R&D Systems Europe Ltd., Abington, UK, AF-410-NA). The antibody solution was then transferred to vial A containing either the 3′-hydroxyl or to vial B containing the 5′-phosphate free oligonucleotides and incubated at 37°C for 3 hours. To end incubation, 1 μL of Stop reagent was added to vial A and B, followed by 30 minutes incubation at RT. To finalize the conjugation, 9.6 μL of the incubation reaction was mixed with storage solution (A or B). The final concentrations of probe A and B were 130 nM. The proximity extension assay (Proseek, Olink, Uppsala, Sweden) procedure was performed according to the manufacturer’s instruction. In brief, 1 μL of sample (Calibrator diluent ± antigen or mouse EDTA plasma ± antigen) was mixed with 3 μL Probe master mix containing probe A, probe B and assay solution. Samples were incubated at RT for two hours. After probe incubation, the samples were transferred to a thermal cycler where pre-extension and extension took place. Until real-time PCR detection, the product was stored at 4°C for a maximum of 24 hours or at −20°C. For the qPCR detection, 4 μL of the extension products was transferred to a 384-well qPCR plate and mixed with 5.8 μL real-time PCR solution and 0.2 μL real-time PCR polymerase (1U/μL). The real-time PCR was run with a denaturation step at 95°C for 5 minutes, followed by 15 seconds denaturation at 95°C and 1 minute annealing at 60°C for 45 cycles. For the Proseek measurements a standard curve of ten-fold dilutions was prepared from recombinant TNF (1 mg/mL) (T7539, Sigma-Aldrich Denmark Aps, Brøndby, Denmark) diluted in Calibrator diluent buffer. This standard curve was additionally mixed with mouse EDTA plasma to investigate the effectiveness of the Proseek TNF measurements in this biologically more complex sample.

### Immunohistochemistry for SP-D in human brain

The study was performed on *post mortem* brain tissue from four stroke patients obtained from the Department of Pathology, Odense University Hospital and the use of human brains was approved by the Danish Biomedical Research Ethical committee for the Region of Southern Denmark (permission number S-20080042). Tissue blocks containing both infarcted and normal appearing brain tissue, in addition to human lung tissue, were embedded in paraffin and serial sections were stained for SP-D, as previously described [9] and for CD45 as routinely performed at the Department of Pathology. The clinical data are provided in Table 1.

**Table 1:** **Clinical data on *post mortem *** **brain tissue from four stroke cases**

**Case**	**Sex/Age of death**	**Infarcted brain area**	**Infarct age**
#1	F/83	Right hippocampus	2 days
#2	M/38	Right temporal lobe	< 5 days
#3	M/57	Left internal capsule	> 7 days
#4	M/59	Right parietal lobe	> 7 days

### Statistical analysis

Results are presented as mean ± SD. For comparison between means in two groups, unpaired Student’s *t*-test was used. For comparisons of more than two groups, two-way ANOVA followed by Bonferroni’s Multiple Comparison Test with the unlesioned control mice as the reference control were used. Pearson’s correlation analysis was used to correlate the percentage of infarcted cortex with TNF mRNA levels. For all tests, *P* < 0.05 was considered significant.

## Results

### Comparable infarct sizes in SP-D KO and WT mice

**Figure 1:**
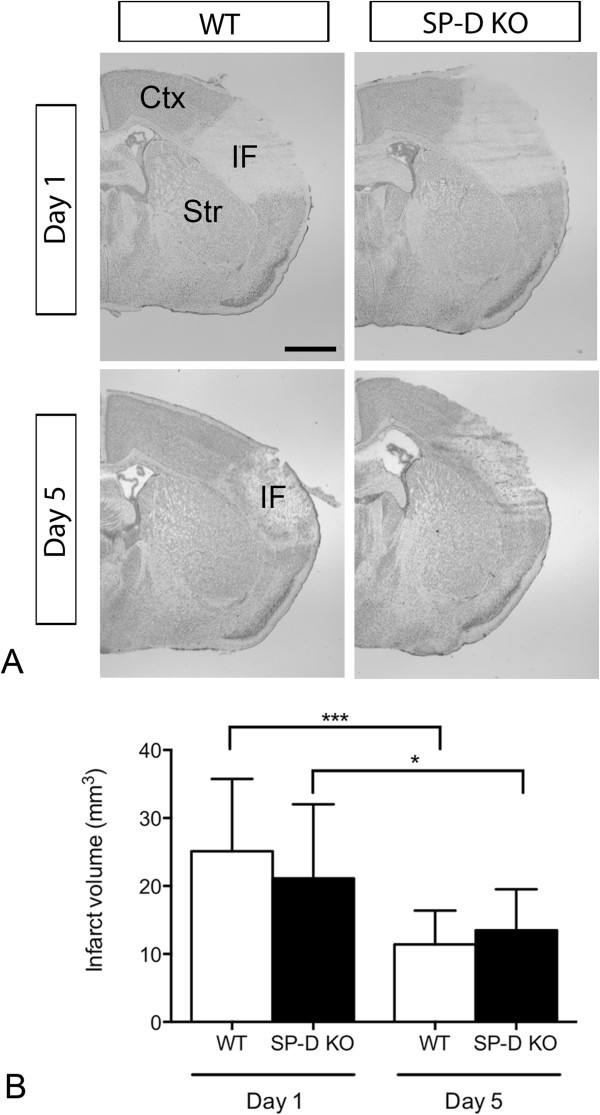
**Infarct development in surfactant protein-D (SP-D) knock out (KO) and wild type (WT) mice after permanent middle cerebral artery occlusion (pMCAO). (A)** Toluidine blue staining of brain sections from WT and SP-D KO mice 1 and 5 days after pMCAO. **(B)** Direct estimation of cortical infarct volume showed no difference in infarct volumes between WT and SP-D KO at 1 or 5 days after pMCAO (Student’s *t*-test; n = 8 to 15). Ctx: cortex, IF: infarct, Str: striatum. Results are expressed as mean ± SD. Scale bar: 1 mm.

Focal cerebral ischemia induced by pMCAO caused a unilateral cortical infarct affecting the frontal and parietal cortices in both SP-D KO and WT mice (Figure 1A). When performing direct infarct volume analysis, we found similar infarct volumes in SP-D KO (21.12 mm^3^ ± 10.89 mm^3^, n = 10) and WT (25.11 mm^3^ ± 10.64 mm^3^, n = 8) mice 1 day after pMCAO (*P* = 0.45, Figure 1B). Similarly, when we corrected for edema at day 1, at the time of maximal brain edema [29], we found that the percentage of infarcted cortex was comparable in SP-D KO mice (35.81% ± 16.48%) and WT mice (38.15% ± 11.28%, *P* = 0.73), emphasizing that SP-D has no influence on acute cerebral infarction. In addition, comparison of infarct volumes in SP-D KO (13.49 mm^3^ ± 6.02 mm^3^, n = 14) and WT (11.43 mm^3^ ± 4.99 mm^3^, n = 15) mice 5 days after pMCAO showed no difference in infarct volumes (*P* = 0.33, Figure 1B). Taken together, the results demonstrate that ablation of SP-D has no effect on the development of the infarct.

### Unaffected TNF response to ischemia in SP-D KO mice

**Figure 2:**
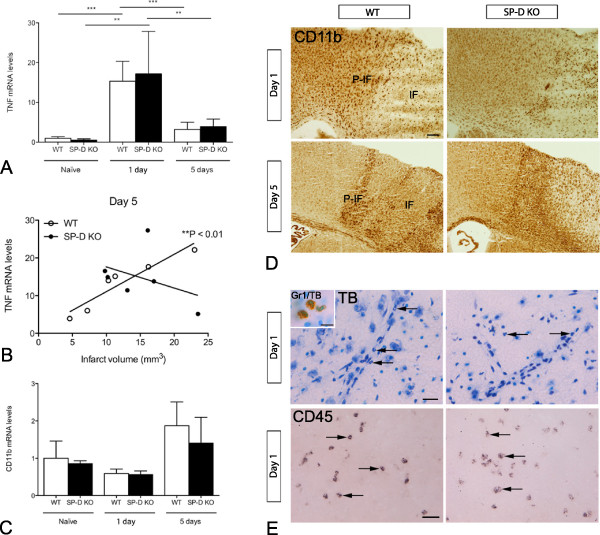
**Changes in inflammatory profile between surfactant protein-D (SP-D) knock out (KO) and wild type (WT) mice after permanent middle cerebral artery occlusion (pMCAO). (A)** No differences were observed in TNF mRNA levels between naïve SP-D KO and WT mice nor between SP-D KO and WT at 24 hours or 5 days after pMCAO. Both genotypes showed a significant increase in TNF mRNA levels at 24 hours compared to naïve and 5 days (one-way ANOVA followed by Tukey’s multiple comparison test, n = 5 to 8). **(B)** TNF mRNA levels correlated with the infarct volume in WT mice 5 days after pMCAO (Spearman’s r = 0.99), a correlation which was not present in SP-D KO mice. **(C)** No difference was observed in CD11b mRNA levels between naïve SP-D KO and WT mice or at 24 hours or 5 days. **(D)** Immunohistochemical staining for CD11b at 1 day and 5 days after pMCAO showed similar microglial-leukocyte activation in SP-D KO and WT mice. **(E)** No difference was observed in PMN infiltration assessed by nuclear morphology in toluidine blue-stained sections, or infiltration with CD45+ cells between SP-D and WT at 24 hours. Insert: immunohistochemical demonstration of Gr1 by PMN. IF: infarct, P-IF: peri-infarct. Scale bars: D = 200 μm, E = 40 μm, insert = 10 μm. **P < 0.01; ***P < 0.001.

In line with previous findings in the C57BL/6 J mouse [30], TNF mRNA levels were significantly upregulated at day 1 (*P* < 0.001), and had decreased again 5 days after pMCAO in WT mice (Figure 2A). The same was true for SP-D KO mice, which also showed significantly increased TNF mRNA levels 1 day after pMCAO (*P* < 0.01), which again were decreased at day 5. Interestingly, in WT mice, we found evidence of a linear correlation between TNF mRNA levels and infarct size at day 5 (Figure 2B), which was not observed at day 1 (*P* = 0.55). This correlation was not observed in SP-D KO mice (day 1: *P* = 0.94; day 5: *P* = 0.44). Overall, however, the results suggest that the TNF response to the acute ischemic attack is preserved in the absence of SP-D.

### Comparable microglial and leukocytic response to ischemia in SP-D KO and WT mice

To our knowledge there is no published information on the effect of SP-D on microglia. SP-D is, however, known to be chemotactic to monocytes and PMNs [1], which both infiltrate the ischemic infarct and express CD11b [31]. To evaluate the effect of SP-D on the microglial and leukocytic response to ischemia we therefore compared brain CD11b mRNA levels in naïve WT and SP-D KO mice and in WT and SP-D KO mice allowed 1 and 5 days survival after pMCAo (Figure 2C). There was no difference in CD11b mRNA levels between WT and SP-D KO mice at any time point investigated and no difference in mice subjected to pMCAO and naïve mice. In line with these findings, inspection of CD11b-stained sections from WT and SP-D KO mice with 1 day (n = 8 and 10 per group, respectively), and 5 days (n = 14 per group) survival (Figure 2D), revealed that the two mouse strains showed a comparable increase of microglial/leukocyte CD11b expression in the peri-infarct and the infarct compared to naïve control WT and SP-D KO mice. To additionally differentiate between leukocyte subsets, we analyzed toluidine blue-stained sections from WT and SP-D KO mice for infiltrating PMNs 1 day after pMCAO (Figure 2E). The use of nuclear characteristics for identification of PMNs was confirmed by staining for Gr1 (insert in Figure 2E), which is expressed on the surface membrane of PMNs and macrophages [36,37]. Infiltrating Gr1+ cells were relatively abundant in the peri-infarct and infarct of WT and SP-D KO mice (Figure 2E), with no difference between genotypes. Similarly, cells expressing CD45, which in addition to macrophages and PMNs is expressed in high levels on lymphocytes [37], showed a comparable infiltration of the peri-infarct and infarct in WT and SP-D KO mice (Figure 2E). Taken together, the findings suggest that ablation of SP-D has no effect on microglial/leukocyte recruitment in response to the ischemic insult.

### Astroglial response to ischemia is comparable in SP-D KO and WT mice

**Figure 3:**
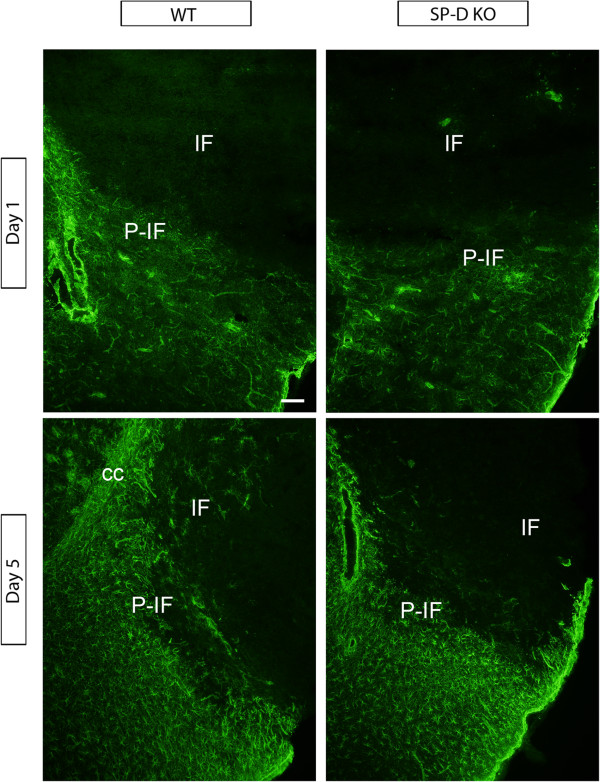
**Astroglial reactions in surfactant protein-D (SP-D) knock out (KO) and wild type (WT) mice after permanent middle cerebral artery occlusion (pMCAO).** Immunofluorescence photomicrographs of the infarct and peri-infarct in SP-D KO and WT mice 1 and 5 days after pMCAO showing similar astroglial response in SP-D KO and WT mice after pMCAO. cc: corpus callosum, IF: infarct, P-IF: peri-infarct. Scale bar: 100 μm.

Differences in astroglial response were assessed by immunofluorescence staining for GFAP. In both SP-D KO and WT mice, GFAP^+^ astrocytes were absent in the infarct core at day 1 (Figure 3). Astroglial GFAP expression was increased in the peri-infarct in both WT and SP-D KO mice 5 days after ischemia as compared to 1 day, with no differences in the strength of the astroglial activation between genotypes (Figure 3). These findings suggest that SP-D has no major effect on the astrocytic response to the ischemic insult.

### Proseek analysis of TNF levels in plasma

**Figure 4:**
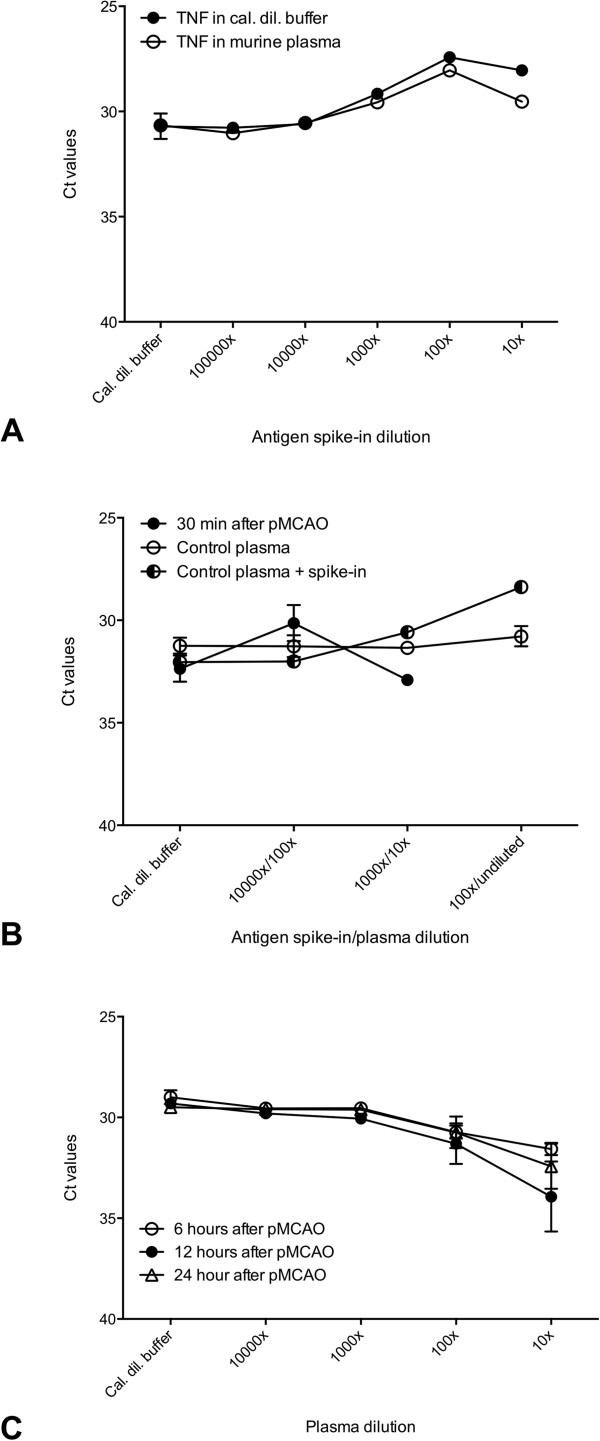
**Proseek analysis of TNF levels in plasma. (A)** The performance of the Proseek TNF assay was evaluated by comparing series of dilution curves of TNF spiked in either Calibrator diluent buffer or mouse EDTA plasma. Recombinant TNF was spiked in Calibrator diluent buffer or mouse EDTA plasma and measured by Proseek TNF assay. The fold dilution is indicated on the x-axis and the signal is plotted as crossing point (Cp) values. The very similar curves illustrate full recovery in plasma, the linear range shows assay efficiency and the hook effect at 10× dilution indicates assay saturation. **(B)** TNF levels in plasma after pMCAO. Plasma samples were obtained 30 minutes after pMCAO and measured in the indicated dilutions (100×, 10× and undiluted). As a positive control to ensure assay performance TNF was spiked in control plasma (Calibrator diluent buffer, 10,000×, 1,000× and 100×) and as negative control plasma from a naïve control mouse (100×, 10× and undiluted). No change in TNF in plasma was observed 30 minutes after pMCAO. **(C)** TNF levels in plasma after pMCAO. Plasma samples were obtained 6, 12 or 24 hours after pMCAO and TNF levels were analyzed at the indicated dilutions (10,000×, 1,000×, 100× and 10×). The TNF level was at background levels and no change in TNF in plasma was observed at the indicated time points after ischemia. Plasma inhibition was introduced in the assay setup, demonstrated by lower signal in the high plasma concentration compared to the buffer level. In (A), (B) and (C) error bars indicate SD.

The Proseek TNF assay performance and recovery was investigated in Calibrator diluent and mouse EDTA plasma. Very similar curves were obtained, indicating a full recovery of the TNF in EDTA plasma samples (Figure 4A). Assay efficacy was demonstrated by the linear range and a so-called hook effect was observed at the highest concentrations (10× diluted), which is in accordance with the proximity extension assay (PEA) [38]. The slightly lower signal obtained in the mouse EDTA plasma could be attributed to a minor plasma inhibition of the antigen spike-in measurements, which was not considered important for the performance of the assay (Figure 4A).The level of plasma TNF was determined in plasma samples obtained from mice 30 minutes, and 6, 12 and 24 hours after pMCAO. The plasma samples obtained 30 minutes after pMCAO were compared with plasma samples obtained from naïve control mice. The plasma TNF level in the control mice was constant at background level. The same low TNF level was observed for the plasma sample taken 30 minutes after pMCAO (Figure 4B). The antigen spike-in dose-response curve was included to ensure assay performance. To ensure that the low level of TNF could not be ascribed to measuring at the incorrect kinetic time point, we investigated plasma from 6, 12 and 24 hours after pMCAO. However, no effect of ischemia on the plasma TNF level was observed, which suggests that induction of permanent focal cerebral ischemia does not affect the systemic level of TNF in young adult mice (Figure 4C).

### Plasma SP-D levels remain low 1 and 5 days after focal cerebral ischemia

Since SP-D is known to be synthesized by Clara cells and type II pneumocytes, in addition to endothelial cells, and potentially could diffuse or be translocated into the circulation, we investigated whether SP-D was present in elevated levels in plasma after pMCAO. Plasma was obtained from mice just prior to decapitation. However, plasma SP-D levels were found to be very low both in naïve WT mice and in WT mice at one and five days after pMCAO (Table 2). This suggests that SP-D levels are not increased in plasma after permanent focal cerebral ischemia in mice.

**Table 2:** Surfactant protein-D (SP-D) levels in plasma

	**Murine SP-D (ng/mL)**	**N**
Control C57BL/6	< 5	8
24 hours C57BL/6 N	< 5	9
5 days C57BL/6 N	< 5	9
24 hours SP-D KO	ND	12
5 days SP-D KO	ND	11

All sample concentrations were below the detection limit (<5 ng/mL).

### SP-D mRNA is expressed in cerebral artery cells in the mouse

**Figure 5:**
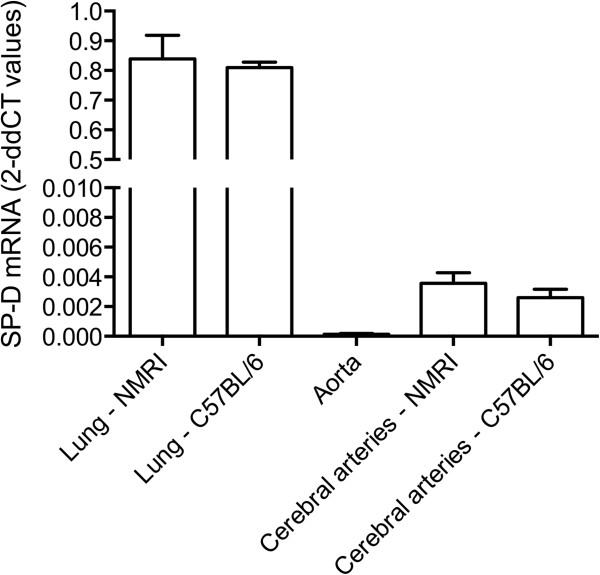
**Surfactant protein-D (SP-D) mRNA expression in cerebral arteries.** Bargraphs visualizing the difference in expression of SP-D mRNA in dissected cerebral arteries from NMRI and C57BL/6 mice compared to lung tissue and aorta. The expression is given in 2dd-Ct values. Results are expressed as mean ± SD.

Next, we investigated SP-D production by brain resident cells. We first investigated whole brain tissue using three different SP-D primer sets. These primer sets were designed to cover different exon-exon junctions of SP-D mRNA. Using this approach, we were able to detect SP-D mRNA in lung and spleen tissue but we did not detect any parenchymal SP-D mRNA expression in neither normal nor ischemic brain (data not shown). Based on previous findings of SP-D protein in endothelial and vascular smooth muscle cells [9,11,12], we then isolated cerebral arteries, including the MCA, from both C57BL/6 mice (n = 2, inbred mouse strain) and NMRI mice (n = 2, non-inbred mouse strain) in order to investigate whether cerebral artery cells produce SP-D mRNA, which is not picked up by whole brain analysis. We found that cerebral artery cells did express SP-D mRNA, which was confirmed using all three primer sets (Figure 5, shown for primer set 3 only). The results using primer set 3 showed that in the lungs from both C57BL/6 and NMRI mice, 2dd-Ct values for SP-D mRNA were 0.81 to 0.84, with 2dd-Ct values in cerebral arteries around 0.003 to 0.004, and for aorta 0.0001 (Figure 5), whereas whole brain 2dd-Ct values were undetectable (data not shown). We found that cerebral artery cells had a 40-fold higher SP-D mRNA level than aortic cells, which have previously been shown to express low levels of SP-D mRNA [9]. In comparison, the level of SP-D mRNA in cerebral artery cells was 200-fold lower than the level in the lung, which is a major site of SP-D synthesis.

### SP-D protein is expressed in vessels in normal appearing and ischemic human brain tissue

**Figure 6:**
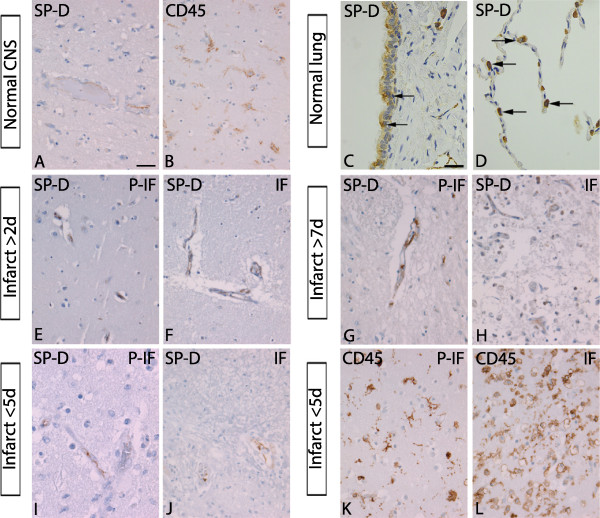
**Surfactant protein-D (SP-D) is expressed in vascular or vessel-associated cells in normal appearing and in ischemic human brain tissue. (A-D)** Immunohistochemical staining showing SP-D in vascular cells (A) and CD45^+^ cells with a microglial morphology (B) in the normal appearing human neocortex. The specificity of the antibody was verified by immunohistochemical staining of (C) Clara cells (arrows) and (D) type II pneumocytes (arrows) in human lung tissue. **(E-J)** SP-D staining was found in vascular and vessel-associated cells in P-IF and IF tissue from human stroke victims with lesions in the right hippocampus (E,F), the left internal capsule (G,H), and the right temporal lobe (I,J). **(K,L)** CD45 staining showing cells with a microglial morphology located in the P-IF (K) and cells with a leukocyte-like morphology in the IF (L) from a patient with a lesion in the right temporal lobe. IF: infarct, P-IF: peri-infarct. Scale bars: (A,B and E-L) = 40 μm and (C,D) = 30 μm.

Since we failed to detect SP-D by immunohistochemistry in murine ischemic brain tissue (data not shown), we turned to human tissue, where SP-D antibodies have been used successfully in our hands [9] (Figure 6). Patient material was chosen in order to cover an early time point (<5 days, n = 2) and a later time point (>5 days, n = 2) after ischemic stroke (Table 1). For comparison, SP-D expression was also analyzed in normal appearing brain tissue from the same tissue blocks, where SP-D immunostaining appeared confined to the endothelium lining the vessels (Figure 6A). In the case of ischemic tissue, we found evidence that SP-D was expressed by vascular or vessel-associated cells in both peri-infarct and infarct in the hippocampus, the temporal lobe, the internal capsule, and the parietal lobe (Figure 6E-J). For verification of the specificity of the primary antibody, Clara cells and type II pneumocytes in the lungs, known to produce SP-D, were confirmed to be SP-D immunopositive (Figure 6C,D). Parallel sections stained for CD45, which is expressed on both microglia and leukocytes, were included to allow us to distinguish between normal appearing brain tissue (Figure 6B), and peri-infarct and infarct core (Figure 6K,L). The results show that SP-D is expressed by vascular or vessel-associated cells both in normal appearing and ischemic human brain tissue.

## Discussion

There is increasing evidence that crosstalk between the immune system in the brain and periphery contributes to determine the long-term neurologic and neuropathologic outcome both in stroke models and in clinical stroke [39,40]. Here we studied the effect of the collectin SP-D, which is involved in systemic inflammation and phagocytosis [41], on cerebral infarction and on post-ischemic inflammatory responses. Our findings of comparable infarct volumes in SP-D KO and WT mice at both one and five days after surgery strongly suggests that SP-D has no effect on cerebral infarction after permanent focal cerebral ischemia; however, the finding does not rule out that SP-D might influence the outcome in reperfusion models of stroke. SP-D mRNA expression in the intact mouse brain was limited to the cerebral vasculature, which is consistent with the initial reports showing very low to undetectable levels of SP-D mRNA in the normal mouse brain [42]. Furthermore, there was no evidence of an upregulation of SP-D mRNA by the parenchymal cells after the ischemic insult. Both sets of observations are consistent with our finding of SP-D expression by vascular or vessel-associated cells both in the normal appearing and ischemic human brain tissue.

In this study, we confirmed the absence of correlation between TNF mRNA levels and infarct volume in the WT mice at one day [30], and found evidence of a linear correlation between TNF mRNA levels and infarct volume five days after induction of ischemia in the WT mice, but not in the SP-D KO mice. The latter observation attracts attention since post-surgical day five coincides with the period of maximal tissue resorption [26,43,44] and maximal expression of plasticity genes [45]. Although, SP-D apparently has no effect of infarct development after induction of permanent focal cerebral ischemia, the finding of an altered TNF response in SP-D KO mice at day five indicates that SP-D modulates the microglial-macrophage TNF response to ischemia in WT mice in the late phase after induction of ischemia. SP-D is known to bind TLR2 and TLR4, as well as SHPS-1/SIRPα that are all expressed on and linked to induction of TNF in microglia and macrophages and involved in stroke [20]. Since microglial-macrophage-produced TNF and SHPS-1/SIRPα are both known to have neuroprotective and pro-regenerative functions [46], SP-D could potentially have indirect effects on post-stroke neural plasticity. Furthermore, since SP-D suppresses the clearance of apoptotic cells by binding to SHPS-1/SIRPα [21], the expression of SP-D by vascular and vessel-associated cells both in the murine and in the human brain might potentially protect the vasculature, while leaving the dying neurons and myelin debris accessible for clearance by the recruited macrophages and microglia. Clarification of the putative role of SP-D in affecting infarct resolution and neural plasticity will require additional experiments, ideally involving the use of models of both permanent and transient focal cerebral ischemia.

Systemic inflammation caused by infection is both associated with increased risk of developing a stroke and with worse outcome after a stroke (review by [40]). A frequent cause of systemic inflammation especially in the elderly is lung infection, which is associated with a massive increase in SP-D in the lung as well as in the circulation [47]. This increase is likely due to translocation of SP-D from the lungs into the vascular system [48]. In this study, we investigated whether surgically induced ischemic injury of the neocortex might result in an increase in SP-D in the circulation. We found that SP-D levels in plasma remained very low, beyond 5 ng/mL, which corresponds to base-line levels [48], up until 1 day after induction of ischemia, arguing against injury to the skull and the neocortex affecting SP-D levels in the circulation. We also monitored plasma TNF in WT mice after ischemia and observed very low levels and no evidence of any ischemia-induced increases up until one day after the surgery. In combination with results by others, who failed to show ischemia-induced increases in plasma TNF in mice two and seven days after transient focal cerebral ischemia [49] this suggests that plasma TNF, like plasma SP-D, is unaffected by the ischemic and surgical injury in young adult mice [46]. This is, in the case of TNF, different from studies of human stroke, where most but not all studies report an elevation of serum TNF from 24 to 72 hours after stroke onset (reviewed in [47]).

Although, we were unsuccessful with the immunohistochemical detection of SP-D in the murine brain, we were able to convincingly show that SP-D mRNA is expressed in higher levels in murine cerebral arteries than in the murine aorta, and that SP-D is expressed in the cerebral microvasculature both in normal appearing and in ischemic human brain tissue. The latter is consistent with our former demonstration of expression of SP-D mRNA in human endothelial and smooth muscle cells, and of a role of SP-D in atherogenesis [9]. This observation is not only relevant to stroke, but also to sporadic Alzheimer’s disease, for which atherosclerosis is a major risk factor.

We conclude that SP-D has no influence on infarct development after permanent focal cerebral ischemia, which is consistent with undetectable production of SP-D by parenchymal cells. The demonstration of SP-D production in cerebral artery cells, however, is consistent with SP-D influencing disease processes in the cerebral vasculature, whereby SP-D may still play a role in stroke pathophysiology, in particular in reperfusion models of stroke.

## Abbreviations

ANOVA: analysis of variance; CAST: Computer Assisted Stereological Test; DAB: diaminobenzidine; ELISA: enzyme-linked immunosorbent assay; GFAP: glial fibrillary acidic protein; HPRT1: hypoxanthine phosphoribosyl-transferase 1; HRP: horse radish peroxidase; KO: knock out; MBL: mannose-binding lectin; MCAO: middle cerebral artery occlusion; OPD: o-phenylenediamine dihydrochloride; PEA: proximity extension assay; pMCAO: permanent middle cerebral artery occlusion; PBS: phosphate-buffered saline; PCR: polymerase chain reaction; PFA: paraformaldehyde; PMN: polymorphonuclear leukocyte; RT: room temperature; sc: subcutaneously; SHPS-1/SIRPα: Src homology 2 domain-containing protein tyrosine phosphatase substrate-1; So-PB: Sorensen’s phosphate buffer; SP-D: surfactant protein-D; TBS: Tris-buffered saline; TLR: toll-like receptor; TNF: tumor necrosis factor; TBS: Tris-buffered saline; WT: wild type.

## Competing interest

The authors declare that they have no competing interests.

## Authors’ contributions

KLL helped design the study, conducted all the animal studies, histological analysis, interpreted the results, performed the statistical analysis and drafted the manuscript. KØ helped design the study, assisted in animal studies, performed histological and brain qPCR analyses, and contributed to the interpretation of the data. BHC helped design the study, conducted all animal studies together with KLL and KØ, performed histological analysis, and interpretation of the data. SH conducted the SP-D ELISA studies. JS and SBT conducted the PLA analysis. MM conducted SP-D qPCR on brain tissue together with KØ. BWK contributed to the human part of the study. PBH contributed to the studies of the mouse cerebral arteries. GLS and BF conceived and designed the study and assisted in writing the manuscript. All authors have read and approved the final manuscript.
